# Rapid access clinic for unexplained lymphadenopathy and suspected malignancy: prospective analysis of 1000 patients

**DOI:** 10.1186/s12878-018-0109-0

**Published:** 2018-08-14

**Authors:** Andrea Kühnl, David Cunningham, Margaret Hutka, Clare Peckitt, Hamoun Rozati, Federica Morano, Irene Chong, Angela Gillbanks, Andrew Wotherspoon, Michelle Harris, Tracey Murray, Ian Chau

**Affiliations:** 1 Department of Medicine, Royal Marsden NHS Foundation Trust, Downs Road, Sutton, Surrey SM2 5PT UK; 2 Department of Computing, Royal Marsden NHS Foundation Trust, London, Surrey UK; 3 Department of Histopathology, Royal Marsden NHS Foundation Trust, London, Surrey UK

**Keywords:** Lymphadenopathy, Rapid access clinic, Cancer diagnosis

## Abstract

**Background:**

In patients presenting with peripheral lymphadenopathy, it is critical to effectively identify those with underlying cancer who require urgent specialist care.

**Methods:**

We analyzed a large dataset of 1000 consecutive patients with unexplained lymphadenopathy referred between 2001 and 2009 to the Royal Marsden Hospital (RMH) rapid access lymph node diagnostic clinic (LNDC).

**Results:**

Cancer was diagnosed in 14% of patients. Factors predictive for malignant disease were male sex, age, supraclavicular and multiple site involvement. Cancer-associated symptoms were present for a median of 8 weeks. The median time from referral to start of cancer therapy was 53 days. Fine needle aspiration (FNA) was performed in 83% of patients with malignancies. Sensitivity and specificity of FNA were limited (50 and 87%, respectively for any malignancy; 30 and 79%, respectively for lymphoma). The vast majority of cancer patients received diagnostic biopsies on the basis of suspicious clinical and ultrasound findings; the FNA result contributed to establishing the diagnosis in only 4 cases.

**Conclusions:**

In conclusion, we demonstrate that Oncologist-led rapid access clinics are successful concepts to assess patients with unexplained lymphadenopathy. Our data suggest that a routine use of FNA should be reconsidered in this setting.

**Electronic supplementary material:**

The online version of this article (10.1186/s12878-018-0109-0) contains supplementary material, which is available to authorized users.

## Background

Peripheral lymphadenopathy has a wide range of infectious, neoplastic and inflammatory differential diagnoses. When assessing patients with unexplained lymphadenopathy, the main challenge is to identify patients with malignancy or other critical conditions requiring urgent specialist care. In addition, the diagnostic work-up should minimize unnecessary procedures and avoid prolonged hospitalization in the interest of cost-effectiveness and patients’ satisfaction.

To optimize management of patients with lymphadenopathy and suspicion of cancer, rapid access lump clinics have been implemented throughout the UK, allowing quick referral routes and close collaboration between hemato-oncologists, radiologists, ENT specialist and surgeons [1]. Similarly, quick diagnosis units have been successfully introduced in other European countries [2] and were recently proposed as suitable diagnostic services for the US healthcare system [3]. However, the optimal set-up of these clinics remains a matter of debate given the difficult task of dealing with a magnitude of distinct conditions in an effective way. Data on performance and outcome of rapid access clinics are therefore essential to further improve this service and to define the most adequate diagnostic pathways for patients with unexplained lymphadenopathy.

Lymphomas are among the most common malignant diagnoses in patients with unclear lymphadenopathy, with the incidence anticipated to rise in the next decades. Lymphomas comprise a heterogeneous group of hematologic malignancies, the most common ones being diffuse large B cell lymphoma (DLBCL), follicular lymphoma (FL) and Hodgkin lymphoma (HL). Lymphoma patients often present with unspecific symptoms commonly seen in non-severe illnesses, which can cause significant delays to specialist referral [4]. Excision biopsy remains the gold standard for diagnosing lymphoma and the full histological work-up requires complex immunohistochemical analyses by an experienced histopathologist. Timely diagnosis and start of treatment is considered particularly important for DLBCL and HL patients who can be cured by multi-agent chemotherapies.

We have successfully established a rapid access multi-disciplinary lymph node diagnostic clinic (LNDC) for unexplained lymphadenopathy at the Royal Marsden Hospital (RMH), a tertiary referral comprehensive cancer centre [5]. Here, we report on the outcome of 1000 consecutive patients referred from 2001 and 2009 to the LNDC with focus on patients diagnosed with lymphoma.

## Methods

We analyzed 1000 consecutive patients referred between September 2001 and September 2009 to the RMH LNDC with available data. The analysis was performed as part of a designated service evaluation for this time period, but results are representative of the current standard in this clinic. The LNDC at RMH was established in 1996 as a rapid access clinic for patients with unexplained lymphadenopathy referred by their General Practitioners [5]. The clinic is held twice a week. The core clinical team comprises a consultant medical oncologist and a lymphoma clinical nurse specialist.

Depending on clinical presentation, diagnostic procedures were arranged as described before [5], including blood tests, microbiology assessments, ultrasound (US), computed tomography (CT) and fine needle aspiration (FNA). There were designated slots for US assessment and results were immediately available. FNA cytology results were available within 1 week and graded C0-C5 as described before [5]. Patients were referred for diagnostic biopsies if suspicion of malignancy was high.

Patients diagnosed with malignancies or benign tumors that required surgical intervention were referred internally to the respective RMH units. Patients with non-malignant conditions for specific treatment were referred to other hospitals as appropriate. Patients with benign reactive lymphadenopathy or self-limiting diseases were discharged from RMH, either immediately or after follow-up clinic visits.

Clinical data, diagnostic results and details on patient management were retrospectively collected on the RMH Electronic Patient Record system. An experienced histopathologist reviewed all lymph node biopsies where a final diagnosis of lymphoma was made. The date of diagnosis for malignant disease was defined as the date of final histological diagnosis. Lymph node areas involved were compared between patient groups using the chi-square test. Multivariate analysis for prediction of malignancy was performed using a stepwise logistic regression model including the following variables: age, gender, ethnicity (white vs. non-white) and site of lymphadenopathy (cervical, axillary, inguinal, extranodal, multiple sites). Data analysis was performed using stata version 13.1.

## Results

### Characteristics and diagnoses of study patients

We analyzed 1000 patients referred between 2001 and 2009 to the RMH LNDC. Patients had a median age of 41 years (range 16–94), were mainly Caucasians (83%) and predominantly female (63%). Malignant disease was diagnosed in 138 (14%) patients (81 with lymphoma (median age 55 years) and 55 with solid tumors (median age 58 years); Table 1). Ninety-one patients had benign neoplasms (median age 50 years) and 89 patients were diagnosed with specific infectious or inflammatory diseases (median age 37 years). The majority of cases (62%) either had reactive/unspecific lymphadenopathy (*n* = 510; median age 37 years), normal tissue/anatomical variants (e.g. prominent muscle; *n* = 46; median age 47 years) or no palpable lesion (*n* = 61; median age 46 years; Table 1). The category reactive/unspecific lymphadenopathy included cases with normal lymph nodes. Of 510 patients with reactive/unspecific lymphadenopathy, 247 (48%) had lymph nodes less than 1 cm in size and 52 (10%) had symptom duration of less than 6 weeks.

**Table 1:** Diagnostic categories

Category	no.
Malignant tumor	138
Lymphoma	81
Other hematological malignancy	2
Solid tumor	55
Benign tumor	91
Non-neoplastic, non-infectious lesion	111
Cyst	47
Vascular malformation	3
Hematoma	4
Normal tissue/variant	46
Goiter	4
Hernia	3
Other	4
Infectious/inflammatory disease	89
Reactive/unspecific lymphadenopathy	510
Nothing palpable	61

Diagnostic categories of the total study cohort (*n* = 1000)

Table 2 shows specific tumor subtypes and infectious/inflammatory diseases diagnosed. Forty-four percent of lymphomas were potentially curable subtypes (HL, DLBCL, Burkitt lymphoma). The most common lymphoma subtype was FL (*n* = 31). Among solid tumors, squamous cell carcinomas (SCC) of the head and neck (*n* = 20) and metastatic melanoma (*n* = 8) were most frequently diagnosed.

**Table 2:** Specific diagnoses

Lymphoma subtypes (no.), *n* = 81	
Hodgkin lymphoma (incl. 1 PTLD)	19
Diffuse large B cell lymphoma	16
Mantle cell lymphoma	4
Burkitt lymphoma	1
Follicular lymphoma	31
Small lymphocytic lymphoma	7
Marginal zone lymphoma	1
Lymphoplasmacytic lymphoma	2
Solid tumors (no.), *n* = 55	
Breast	6
Head and neck (SCC)	20
Thyroid	1
Salivary gland	4
Upper GI adenocarcinoma (1 oesophageal, 1 gastric)	2
Cervix/ovarian	2
Skin (8 Melanoma, 1 SCC, 1 Merkel cell)	10
Prostate	1
Lung (non-small cell)	1
Renal	1
Olfactory neuroblastoma	1
Unknown Primary	6
Benign tumors (no.), *n* = 91	
Angioma/angiofibroma	2
Lipoma	44
Fibroadenoma	6
Pilomatrixoma	2
Pleomorphic salivary gland adenoma	15
Warthin’s tumor	18
Granular cell tumor	1
Follicular adenoma (thyroid)	1
Schwannoma	1
Not specified	1
Infectious/inflammatory diseases (no.), *n* = 89	
Chronic sialadenitis/tonsillitis	12
Dermatopathic lymphadenopathy	12
Local inflammation/abscess	8
Sarcoidosis/granuloma	13
Kikuchi’s disease	2
Specific acute infections	42
Toxoplasmosis	14
Tuberculosis	13
Epstein-Barr virus	6
Human immunodeficiency virus	5
Syphilis	1
Mumps	1
Bartonella infection	2

PTLD indicates post -transplant lymphoproliferative disorder; SCC indicates squamous cell carcinoma

Specific diagnoses assigned to patients on the study (*n* = 1000)

### Clinical presentation of cancer patients

All patients diagnosed with malignant disease presented with nodes/lumps of more than 1.5 cm in size. Cancer-associated symptoms were present for a median of 8 weeks (range 0–832). Of 35 patients with HL or DLBCL, 15 (43%) had elevated LDH levels and 5 (14%) had B-symptoms as indicators for highly proliferative disease. Median duration of symptoms in these patients was 7 weeks (range 1–104).

Patients with malignant disease presented significantly more often with supraclavicular nodes and lymphadenopathy involving multiple sites compared to non-malignant cases (Table 3). Frequency of extranodal involvement was significantly lower in malignant vs. non-malignant conditions (*P* = 0.009). Axillary involvement was more frequently seen in solid tumors as compared to lymphomas (16% vs. 5%; *P* = 0.03). Lymphomas presented significantly more often with multiple site involvement (22% vs. 5%; *P* = 0.008) in comparison to solid tumors.

**Table 3:** Anatomical areas involved

Areas involved	Malignant (*n* = 138) no. (%)	Non-malignant (*n* = 862) no. (%)	*P*
Cervical	71 (51)	518 (60)	0.055
Supraclavicular	11 (8)	26 (3)	0.004
Axillary	13 (9)	119 (14)	0.158
Inguinal	19 (14)	77 (9)	0.073
Multiple sites	21 (15)	47 (5)	< 0.001
Extranodal	3 (2)	75 (9)	0.008

Sites of nodal- and extranodal involvement in malignant and non-malignant cases

In multivariate analyses, supraclavicular and multiple site involvement were significantly predictive of malignancy, whereas extranodal involvement was an independent predictor of non-malignant disease (Table 4). In addition, higher age and male sex were predictive of malignant disease.

**Table 4:** Multivariable analysis for prediction of malignant disease (*n* = 1000)

Variables	OR	95% CI	*P*
Age (years)	1.04	1.03–1.06	< 0.001
Male sex	2.84	1.92–4.21	< 0.001
Supraclavicular	2.41	1.10–5.31	0.03
Multiple sites	4.02	2.20–7.34	< 0.001
Extranodal	0.17	0.05–0.58	0.004

*OR* indicates odds ratio, *CI* indicates confidence interval

### Cancer treatment and waiting times

Time to final diagnosis and treatment was assessed in 122 cancer patients with available data. The median time from first clinic visit to full histological diagnosis was 22 days (range 1–924), 28 days for lymphomas (range 7–356) and 19 days for solid tumors (range 1–924). The main reason for diagnostic delays in lymphoma cases was presence of mild/intermittent symptoms, leading to prolonged monitoring of patients before a diagnostic biopsy was arranged.

Seventy-five (56%) patients with malignant disease received systemic treatment, 19 (14%) received initial radiotherapy and 20 (15%) primary surgery. Four patients with metastatic solid tumors received best supportive care only. In 16 patients diagnosed with indolent lymphoma a watch and wait policy was adopted. The median time from referral to start of therapy was 53 days (range 21–930; 56 days for lymphomas and 50 days for solid tumors), including watch and wait and best supportive care as therapies. After exclusion of cases delayed due to patients’ decision, the median times to therapy were 50 days (lymphomas) and 48 days (solid tumors).

Rapid and streamline pathways for diagnosis and therapy might be of particular importance for potentially curable lymphomas, such as HL and DLBCL. Median time from referral to start of therapy in HL was 48 days (range 26–202) and 45 days for DLBCL (range 22–116). Median time from referral to start of therapy has improved since implementing specific cancer waiting time targets in the UK in 2005, with a median time of 38 days between 2005 and 2009 compared to 51 days between 2001 and 2004 (Additional file 1: Figure S1).

### Diagnostic procedures

96% of patients with malignancy and all lymphoma patients had a core or excision biopsy as diagnostic procedure. Six solid tumor cases were diagnosed with FNA only (5 did not have a biopsy, 1 had a non-diagnostic biopsy). FNA was performed in 423 of 1000 cases, in 114/138 (83%) of malignancies and in 63/81 (78%) of lymphomas.

FNA results were inadequate (C0/C1) in 94/423 (22%) of patients, which was similar with US guidance [68/316 (22%)] and without [26/107 (24%)]. FNA raised suspicion of cancer (C3–5 cytology) in 57/114 (50%) of all malignancies and in 19/81 (24%) of lymphomas. Benign cytology (C2) was seen in 44/114 (39%) malignancies, the majority of which were lymphomas (*n* = 39). Sensitivity of US and FNA was 93 and 50% to detect any malignancy, and 88 and 30% to detect lymphoma, respectively. Specificity was 96% (US) and 87% (FNA) for detection of all malignancies, and 91% (US) and 79% (FNA) for detection of lymphoma.

Given the limited sensitivity and specificity of FNA as diagnostic tool, we analyzed which diagnostic finding actually prompted the LNDC team to arrange a biopsy in malignant cases (*n* = 133; Table 5). Most patients (*n* = 129) were referred to biopsy on the basis of high clinical suspicion with or without additional abnormal findings in US performed on the day of clinic visit. Only in 2 cases with cancer of unknown primary and 1 Burkitt lymphoma patient, the FNA C5 result led to referral for biopsy. In 1 patient with lymphoplasmacytic lymphoma, the FNA result (C3) was the only finding that raised suspicion of malignancy and prompted a diagnostic biopsy. In 10 patients with solid tumors (5 head and neck, 3 breast, 1 salivary gland, 1 unknown primary), the FNA C4/5 result was important to decide on doing a core rather than excision biopsy. In 11 patients with minor symptoms (10 lymphoma cases and 1 salivary gland carcinoma), the diagnostic biopsy was delayed due to FNA C2 results, with a median time from first visit to diagnosis of 79 days (range 47–212). Thus, FNA was of limited value for establishing the diagnosis in the majority of malignant diseases in our series.

**Table 5:** Indication for biopsy in malignant cases (*n* = 133)

Indication for biopsy in malignant cases	Reason for US/FNA not impacting on decision-making
Clinical suspicion (*n* = 68)	No US performed (*n* = 63)
	US inconclusive/not suspicious (*n* = 5)
	No FNA performed (*n* = 19)
	FNA results inconclusive/not suspicious/not awaited (*n* = 49)
Clinical and US suspicion (*n* = 61)	No FNA performed (*n* = 5)
	FNA results inconclusive/not suspicious/not awaited (*n* = 56)
Clinical suspicion and FNA C5 (*n* = 2)	No US performed (*n* = 2)
FNA C3 result (*n* = 1)^a^	No US performed (*n* = 1)
Clinical and US suspicion, FNA C5 (*n* = 1)	Not applicable

^a^No clinical suspicion of malignancy

Diagnostic findings that led to performing a confirmatory biopsy in patients with malignant disease (*n* = 133)

Most non-malignant cases [539/862 (63%)] did not have either biopsy or FNA. Among 310 non-malignant cases undergoing FNA, 81 (26%) were inadequate, 190 (61%) showed benign cytology and 13 (4%) raised suspicion of malignancy. In 26 cases the FNA result was diagnostic for the non-malignant condition (11 pleomorphic salivary gland adenomas, 6 Warthin’s tumors, 3 branchial cysts, 2 tuberculosis cases, 1 abscess, 1 toxoplasmosis, 1 hematoma, 1 pilomatrixoma). Biopsies were not performed in 742/862 (86%) of non-malignant cases, mainly guided by clinical examination and US (Additional file 1: Figure S2). In 152/742 (20%) cases with non-malignant disease, the FNA result helped to establish the diagnosis and to avoid a more invasive biopsy.

## Discussion

Implementation of rapid access diagnostic clinics for patients with unexplained lymphadenopathy facilitates early diagnosis of cancer. Here, we provide a detailed report on 1000 consecutive patients seen in the multidisciplinary LNDC at RMH between 2001 and 2009.

The pick-up rate for malignant diseases in our study was 14% which is in line with previous findings at our [5] and other institutions [6] and similar to results from neck lump clinics [7–10]. However, a recent Spanish study investigating 372 patients with unexplained lymphadenopathy referred from primary health care centers to an internist-led quick diagnosis unit reported a cancer rate of 32% [2]. The study excluded patients without palpable lesion at the time of clinic visit, which was found in 6% of our cases. In addition, they had a lower incidence of reactive nodes (42%) and only 4 cases with benign tumors. In contrast, reactive and benign findings accounted for 71% of referrals in our series. Given the set-up of our clinic in a tertiary care cancer center involving assessment by a consultant medical oncologist and a lymphoma clinical nurse specialist, a better selection of patients who require urgent assessment would be preferable.

Most cancer referral guidelines suggest referral of patients with unclear lymphadenopathy or lumps greater than 1-2 cm in size [1, 11]. Our findings support adherence to a minimum size limit. Half of reactive nodes but no case of malignancy presented with sub-centimeter lymphadenopathy in our cohort. The use of calipers for exact nodal measurements might help to increase the quality of referrals. In addition, extranodal lumps (not in the breast or head and neck) that are unchanged in size for many years should not be subject to an urgent referral pathway.

Our study shows that by far the most important diagnostic tool is examination/evaluation by an experienced clinician, taking into account several factors to estimate the probability of malignancy, such as size, texture and site of the lump, symptoms, risk factors, and examination of loco-regional and disease-related sites. The majority of non-malignant cases did not undergo any invasive investigation and could therefore efficiently be assessed outside a specialist center. Thus, some form of local Hemato-oncology involvement or remote Specialist triaging, alongside improved communication and guidance between primary care and specialist centers might further assist appropriate patient selection. This would allow for a more effective use of resources and would spare low-risk patients the psychological burden of being assessed for cancer.

We and others have identified clinical factors in patients with unexplained lymphadenopathy that predict for malignancy [5, 6] Here, we validate our previous findings identifying male sex, age, as well as supraclavicular and multiple site involvement as independently predictive for malignant disease. Presence of these features in patients with unexplained lymphadenopathy should alert clinicians to the possibility of underlying cancer.

Referrals of patients with lymphomas and solid tumors occurred in a timely manner with median symptom duration of only 8 weeks at the time of presentation in our clinic. Particularly lymphoma-related symptoms are often not indicative of malignant disease or serious illness, which usually leads to a longer time to seeking medical help compared to other cancers [4]. Howell et al. [12] reported a median time of 10 weeks from onset of lymphoma-related symptoms to seeking medical help and Summerfield et al. [13] observed a mean time of 16 weeks. The shorter intervals in our series might indicate an increasing public awareness about the need to investigate an unexplained change in health. Referred patients were seen promptly in our clinic (mean waiting time of 7 days). A median time from referral to start of treatment of 53 days in patients diagnosed with cancer still warrants improvement. Similar to previous findings [12], main delays occurred after the first clinic appointment, and were predominantly “diagnostic delays” for lymphoma patients (mainly indolent lymphomas) and “therapy delays” in solid tumors (mainly palliative treatment).

Indolent lymphomas typically present with diffuse, mild symptoms and have a high false-negative rate in FNA assessment. Accordingly, the main reason for delay in the diagnosis of indolent lymphomas was an initially observational approach, in some cases supported by benign FNA results. To diagnose indolent lymphomas quicker, cases without high clinical suspicion of malignancy would generally have to undergo early biopsies. This would significantly increase the rate of invasive procedures for non-cancer patients.

FNA assessment on the day of clinic visit in patients with suspicion of malignancy is part of our LNDC set-up. This is in line with national recommendations for rapid access head and neck lump clinics. However, lymphomas are usually the most common malignancies diagnosed in clinics for general lymphadenopathy or head and neck lumps and FNA is not regarded an appropriate tool for lymphoma diagnosis. Limitations of FNA in lymphoma are well documented [14–16]. Our findings further support these data with the sensitivity of FNA being only 30%. Accuracy of FNA is better for solid tumors, but significantly depends on the exact site of disease as shown for head and neck lumps [16]. A rapid access LNDC deals with a variety of cancer types and routine use of FNA for every patient might not be optimal in this regard.

FNA as primary diagnostic tool should have a minimal rate of inadequate and false-negative samples and should decrease the need for diagnostic biopsies. This however highly depends on the clinic setting: local FNA performance, clinical expertise, availability/quality of US assessment and frequency of cancer types. The utility of FNA is certainly higher in cytologist-led one-stop clinics were samples can be immediately assessed and retaken if necessary. In addition, accuracy of FNA for lymphoma, particularly high-grade NHL, can be improved by flow cytometry [17, 18], but this is an expensive technique only available at specialist centers. Of note, even if FNA is indicative/diagnostic for lymphoma, this will not substitute for a surgical biopsy to allow the full range of tests needed for exact diagnosis. Also in solid tumors, the majority of patients still require a biopsy after FNA to establish the diagnosis and to provide sufficient material for molecular and immunohistochemical testings. In view of the increasing use of targeted therapies, the need for adequate tissue to assess predictive biomarkers will further grow. In our series, only 5 patients with solid tumors were diagnosed with FNA only. In a further few cancer cases, FNA results were important to decide on performing a biopsy. In the majority of malignant cases, indication for biopsy was guided by clinical suspicion and US features. In addition, many patients required repeat aspirations (data not shown), which involves additional costs and follow-up visits. Repeat false-negative results (including inadequate samples) are not only misleading for clinicians, but also impose significant stress on patients.

US is highly sensitive to distinguish malignant from non-malignant lumps and can provide important information about the type of malignancy. US is cheap, safe and widely available. Our data indicate that if an integrated US service and a highly experienced clinical team is available, pre-selection of patients for FNA should be more stringent. For example, FNA should be performed if there is high suspicion of SCC of the head and neck. Or, if there is low suspicion of malignancy and a benign FNA result is regarded sufficient to rule out malignancy. On the other hand, FNA should be avoided if lymphoma is suspected (e.g. age, B-symptoms, typical US features).

## Conclusions

In conclusion, this is the largest dataset of a rapid access clinic for patients with unclear lymphadenopathy. Our results provide valuable insights into the successful performance of our LNDC and build a basis for further improvement of this diagnostic service model.

## Additional file

Additional file 1 : **Figure S1**. Timelines for diagnosis and therapy in HL and DLBCL. Timelines from referral to start of therapy for HL and DLBCL patients diagnosed between 2001 and 2004 and between 2005 and 2009. **Figure S2**. Diagnostic procedures used to decide against the need for a biopsy in non-malignant cases. Relevance of clinical assessment, ultrasound, FNA and CT scan in the decision-making to avoid a biopsy in 742 non-malignant cases. (DOCX 45 kb)

## Abbreviations

CT: Computed tomography
DLBCL: Diffuse large B-cell lymphoma
FL: Follicular lymphoma
FNA: Fine needle aspiration
HL: Hodgkin lymphoma
LDH: Lactate dehydrogenase
LNDC: Lymph node diagnostic clinic
RMH: Royal Marsden Hospital
SCC: Squamous cell carcinoma
US: Ultrasound
