# Supplementary material for: Rapid access clinic for unexplained lymphadenopathy and suspected malignancy: prospective analysis of 1000 patients
Source: BMC Hematol. 2018 Aug 14;18:19. doi: 10.1186/s12878-018-0109-0 (PMC6092787; doi:10.1186/s12878-018-0109-0)
Supplement: Supplementary file 1 — : Figure S1. Timelines for diagnosis and therapy in HL and DLBCL. Timelines from referral to start of therapy for HL and DLBCL patients diagnosed between 2001 and 2004 and between 2005 and 2009. Figure S2. Diagnostic procedures used to decide against the need for a biopsy in non-malignant cases. Relevance of clinical assessment, ultrasound, FNA and CT scan in the decision-making to avoid a biopsy in 742 non-malignant cases. (DOCX 45 kb) [file 12878_2018_109_MOESM1_ESM.docx]

**Additional Files**

**Additional Figure S1: Timelines for diagnosis and therapy in HL and DLBCL.**

**Add. Figure S1: Timelines for diagnosis and therapy in HL and DLBCL.** Timelines from referral to diagnosis (grey) and from diagnosis to start of therapy (red) are shown for each HL and DLBCL patient diagnosed between 2001-2004 and between 2005-2009 (UK Department of Health target for 2005: 62 days from urgent GP referral for suspected cancer to first definitive treatment for all cancers).

**Additional Figure S2: Diagnostic procedures used to decide against the need for a biopsy in non-malignant cases (n=742)**

| Clinical | N=323 |  |  |  | |
| --- | --- | --- | --- | --- | --- |
| Clinical | US | N=239 |  |  | |
| Clinical | FNA | N=42 |  |  | |
| Clinical | US | FNA | N=109 |  | |
| Clinical | US | CT | N=10 |  | |
| Clinical | US | CT | FNA | N=5 |  |
| FNA^a^ | N=1 |  |  |  | |
| CT^b^ | N=13 |  |  |  | |

^a^FNA diagnostic for benign tumor after high clinical suspicion of malignancy; ^b^Normal CT scan after high clinical suspicion of malignancy
